# Book Review

**Published:** 1886-03

**Authors:** 


(1) *Handbook of the Diseases of the Nervous System.* By JAMES ROSS, M.D., LL.D. London: J. & A. Churchill, 1886. Pp. 723.

(2) *Rome in Winter and the Tuscan Hills in Summer.* By DAVID YOUNG, M.D. London: H. K. Lewis, 1886. Pp. 288.

(3) *An Introduction to Practical Bacteriology.* By EDGAR M. CROOKSHANK. London: H. K. Lewis, 1886. Pp. 249.

(4) *Surgical Operations. Pt. I., Ligature of Arteries.* By Sir W. MACCORMAC, F.R.C.S. London: Smith, Elder & Co., 1885. Pp. 138.

(5) *The Influence of Sex in Disease.* By W. ROGER WILLIAMS, F.R.C.S. London; J. & A. Churchill, 1885. Pp. 39.

(6) *Operative Surgery of the Human Brain.* By JOHN B. ROBERTS, M.D. Philadelphia: Blakiston & Son, 1885. Pp. 80.

(7) *The Insane in the United States and Canada.* By D. HACK TUKE, M.D., LL.D. London: H. K. Lewis, 1885. Pp. 242 and Appendices.

(8) *Atlas of Venereal Diseases.* Fasc. I., II., III., IV. By P. H. MACLAREN, M.D. Edinburgh: Young J. Pentland, 1885.

(1) Dr. Ross's *Handbook on the Nervous System* deserves a more extended notice than we can give to it. However, his reputation as a neurologist and as a bookmaker is so well established that anything we can say will scarcely add to it. This work is a condensation of his large treatise, intended for the use of students and for the "busy practitioner." As coming from Dr. Ross's pen the work can scarcely be less than authoritative. It is, besides, clear, succinct, and readable. The descriptions are always graphic—sometimes almost photographic. The treatment, naturally, is not always enthusiastic, but it is always as definite as circumstances permit. The classification, though clinical, is easily followed, and precise enough. The work is altogether good—too good, we fear, for the continuance of the prosperity of his large work.

The excellence of the get-up of the book, its binding, printing, and illustrations, are beyond all praise. Our English conceit was however somewhat lowered by the discovery on the back of the title page of the name of a Philadelphia printer. The printing in America, and the consequent insufficiency of proof reading, may explain the long list of "errata and important omissions."

(2) Reading Dr. Young's book in English mid-winter is almost as good as taking a holiday. We readily and heartily admit that the climate of Rome is not as bad as it is painted, and our only regret is that we cannot be there to verify Dr. Young's statements. That many of the risks of residence in *Rome in winter* are from ignorance, carelessness, or exaggeration we cannot gainsay; we must confess that Dr. Young's description of *the Tuscan Hills in summer* is excessively tempting. Recreation and sunlight are therapeutics; we must thank Dr. Young for giving us in these respects at once a guide and a treatment, a physician and an apothecary.

The dangers of the Roman climate have undoubtedly been overstated; the advantages of residence among the Tuscan Hills have not been fully described. Dr. Young's work will help to remedy misconceptions in both directions.

(3) Some knowledge of *Bacteriology* is now becoming essential to the practitioner of medicine; and no doubt a course of instruction in its methods will soon become a necessary and integral part of the student's curriculum. In the meantime, as our knowledge of the subject is crystallising into definiteness, we gladly welcome any clear and straightforward account of such facts concerning Bacteria as have been settled, and such methods of study as have been approved best. Mr. Crookshank has evidently spared no pains in making himself acquainted with the work of our most distinguished Bacteriologists, in their laboratories as well as in their writings. His descriptions of the methods of manipulation and culture are short and clear, and an ordinary student or clinical worker with some training in physics and microscopy will find no difficulty in following them in practice. The work covers a wide field, but from the excellent system and classification followed it is easy to select and separately study any one organism or group of organisms.

The book is abundantly and beautifully illustrated.

(4) So much has already been written upon the *Ligature of Arteries* that conspicuous merit alone would justify the appearance of a new work on the subject. Sir William MacCormac's book gives an excellent account of the anatomical and practical surgery of arteries, and it is thoroughly well illustrated. The language is clear and not superabundant; the matter is probably sufficient for students, though we think a little more space might have been spared from other less important parts to the description of materials for ligature. The drawings by Mr. William Anderson are of a very high order. As it seems necessary that fingers holding knives and pinching up fasciæ and tying ligatures should be drawn, it is a great comfort to have them drawn artistically. The anatomical drawings are inferior to none we have seen.

(5) Mr. Roger Williams has laboured hard in his attempt to throw light on the subject of *The Influence of Sex in Disease.* As a matter of fact the elaborate statistics provided do not range over disease as applicable to the human species, or even to the inhabitants of England, but only to disease as limited to the class of persons who attend London Hospitals. In this limited sense they will no doubt prove of value. In handling his figures Mr. Williams is occasionally open to criticism, and a few fallacies of reasoning might be pointed out.

(6) Dr. Roberts has done good service to surgery by writing this small work on the *Operative Surgery of the Human Brain.* His work is the first real attempt to bring the literature of the subject abreast of the enlightened practice of the day. The author is constructive as well as destructive. Not only does he uproot traditional fallacies and general misconceptions: he boldly lays down clear and definite lines of treatment and minutely elaborates rules for diagnosis. The book is a model of its kind; concise and full, learned and practical, conservative of proved truths, and pushing research into new channels.

(7) Dr. Hack Tuke's work is a painful commentary on the state of certain *Lunatic Asylums in the New World.* The effects of his fearless and just criticism are already beginning to have good result. His historical disquisitions are full of interest, and the special descriptions of special institutions must be of great value to the alienist.

(8) Dr. Maclaren's *Plates of Venereal Diseases* are probably as good as it is possible to make them: we have seen none better of British production. The letterpress is general as well as special; it includes a short account of the disease under consideration, as well as of the condition depicted in the drawing. The work so far is one of conspicuous merit.

### FOUR OF CASSELL'S MANUALS.

(1) *Comparative Anatomy and Physiology.* By F. Jeffrey Bell. Pp. 555.
(2) *Surgical Diseases of the Kidneys.* By H. Morris. Pp. 548.
(3) *Surgical Diseases of Children.* By Edmund Owen. Pp. 518.
(4) *Fractures and Dislocations.* By T. Pickering Pick. Pp. 524.

The enterprise of Messrs. Cassell in medical publishing is undoubtedly proving a success. The object of their series of manuals, "to present to the Practitioner and Student of Medicine original, concise, and complete monographs on all the principal subjects of medicine and surgery," is on a fair way to being fulfilled. Of the manuals already in print scarcely one descends to mediocrity; some are of a high order of excellence; and two or three are probably the best monographs in existence on the subjects which they deal with. The books are printed in large clear type on good paper; they are of "a size convenient for the pocket" (what pocket, by the way? and how many of us do or ever did carry medical books in our pockets?), and are bound substantially in limp cloth. Finally, they are, as compared with most medical works, cheap.

We feel—we are not sure whether we hope or we fear—that these manuals inaugurate an era in medical bookmaking. It is not so very long ago since the whole science and art of medicine could be purchased in one moderately-sized volume, the work of one man. In our own generation we have seen the stupendous efforts of individuals productive of moderately good works, embracing the whole of surgery or the whole of medicine; but a future generation will almost certainly not see this. Only cyclopædias will contain the knowledge. It is merely a question whether the cyclopædias shall be bound together in volumes to be purchased only in bulk, or bound separately in monographs and purchased in detail. Whatever advantages may pertain to the latter mode of production we may find in Cassell's series.

(1) Professor Jeffrey Bell, in his manual of *Comparative Anatomy and Physiology*, has admirably performed his part. In his descriptions, by grouping together organs rather than animals, he has succeeded in giving a coherent account of function as well as form. It is true that a complete comparative physiology does not yet exist; but by adroitly welding together our more complete knowledge of anatomy with our less complete knowledge of physiology, he has been able to make us forget our deficiencies in the one, while he has greatly added to our interest in the other.

The manual is pre-eminently a student's book—clear and simple, and definite in language and arrangement. It is well and abundantly illustrated, and it is "readable" and interesting. On the whole, we consider it the best work at present existing in the English language to place in the hands of the medical student.

(2) We confess to having been somewhat disappointed with Mr. Morris's work on *Surgical Diseases of the Kidneys*. Perhaps it was that we expected too much. Knowing what Mr. Morris has already done for the surgery of the kidney, we expected that in this work he would have taken up and held in his own hands the guiding reins of renal surgery for years to come. We have read and re-read the work, and we have finally laid it down with the impression that it might be surpassed. It is a peculiarity of the work—not unknown in the work of men who are modest—that in those parts where Mr. Morris is known most to excel he is weakest; while in other lines of study, which happen also to be of less importance and interest, he is strongest. The surgery of the kidney is crowded out by abnormalities and malformations; personal instruction is replaced by bibliography; and practice is ousted by theory. No doubt it is all valuable; we can scarcely get the same amount of trustworthy information so well laid down anywhere else; but it is not well balanced for the ends which a clinical manual is supposed to serve. The last chapter, of seventeen pages, contains the "methods of performing the operations on the kidneys." Surely in a work of over 500 pages on the *Surgical Diseases of the Kidney*, this is not enough. Chapter XIII., on "Injuries of the kidney communicating with an open wound,"—not a large subject—is longer. This is our only complaint—want of proportion. The first half of the book ought to be cut down, and the second part expanded.

There is abundant evidence everywhere of wide reading and accurate knowledge. The work is one which, now that we have it, we should not like to be without; but when another edition is called for, which we hope may be soon, we shall be glad to see some improvements in the direction which we have indicated.

(3) Sound common sense and graphic language are the most prominent attributes of Mr. EDMUND OWEN'S book on *The Surgical Diseases of Children*. The writer knows what he is about, and there is no possibility of being in doubt as to the meaning he wishes to convey. It is no easy task to write a book on the surgical diseases of children. All classes of practitioners claim an interest in children. The midwife; the man who calls himself gynæcologist, who deals in diseases of women, and makes excursions into the abdominal cavities of men; the pure physician; and lastly, the ubiquitous throat specialist, all trench on the domain of children. Take croup and diphtheria, for instance. The disease and the operation for it are described in books of surgery, in books of medicine, in special works, and in the monographs of specialists on the mouth, nose, and throat. Syphilis, rickets, nævus, and the crowd of congenital deformities—what is their province, and where are they not described? Mr. Owen has legitimately got hold of them all in his book, and he has handled them all well.

The work is one of personal experience honestly recorded, and this is its conspicuous merit. We may not believe that in every possible detail the practice of Mr. Owen is the very best, but we cannot fail to admit that everywhere his practice is good. The operation for extroversion of the bladder is very summarily treated; he is not very strong on knock-knee; and, graphic as is his description of tracheotomy, we are not sure that it is absolutely perfect. For the last operation an incision of one and a half to two inches in an infant is surely an outcome rather of the spirit of the anatomical dissector than of the surgical operator. The writer errs on the safe side; it is right that his instructions are rather to the average practitioner and the young resident officer than to the skilled surgeon.

We heartily commend Mr. Owen's work to our readers.

(4) The work of Mr. PICK on *Fractures and Dislocations*, though good, is considerably behind the best in the series. In the main it is trustworthy, but it is not everywhere up to date; it is nowhere original, and not always discriminative. It is far behind Hamilton's classic, scarcely superior to Stimson's work, and certainly inferior to more than one article in the cyclopædias and dictionaries.

As to his practice, there is little to be objected to; it is nearly always good; if he had made a tour of the provincial hospitals before putting it on paper, it would have been better. It is on the old lines, clogged with effete customs. Plans which he has evidently never seen he condemns on false grounds; others which he has never heard of he, of course, ignores. In practice the book has all the merits and all the drawbacks of being almost purely personal.

In theory he distinctly fails. How is it possible to satisfy the requirements of science by speaking of the union of fractures under the three heads (p. 31)—(1) "Transverse fracture," (2) "More or less oblique fracture," and (3) "Fracture in which the ends of the bone are not kept strictly in apposition"? His remarks on "osteomyelitis" as a result of compound fracture are, to say the least, not up to date (p. 74). The spirit of the independent thinker is conspicuously absent in the important subject of fractures of the neck of the thigh bone (p. 243), and in many other subjects besides. In theory the work suffers from the want of independent and original work. It fills a gap in Cassell's series; it adds little to the sum total of surgical knowledge.

